# Supplementary material for: Spatial price transmission in the onion markets of Bangladesh: An application of NARDL approach
Source: PLoS One. 2023 Apr 19;18(4):e0284555. doi: 10.1371/journal.pone.0284555 (PMC10115283; doi:10.1371/journal.pone.0284555)
Supplement: Attachment — Submitted filename: Respose to Reviewers.docx [file pone.0284555.s002.docx]

# Title: Spatial Price Transmission in the Onion Markets of Bangladesh: An Application of NARDL Approach

We express gratitude to the reviewers and editors for providing the valuable comments to improve our paper. We have rewritten each section of the paper thoroughly according to both the reviewers’ and editors’ comments. We hope that the changes made in the revised version (as shown below) will be acceptable. All changes have been highlighted (yellow colour) in the manuscript.

**Editor and Reviewer Comments**

# COMMENTS TO AUTHORS’ SPECIFIC RESPONSES

| **Editors and Reviewer Comments** | **Author’s Response** |
| --- | --- |
| 1. Please ensure that your manuscript meets PLOS ONE's style requirements, including those for file naming. | 1. Thanks to the editors for their valuable comments. Now, we have followed the PLOS ONE’s template and file naming style. |
| 2. Please provide additional details regarding participant consent. In the ethics statement in the Methods and online submission information, please ensure that you have specified (1) whether consent was informed and (2) what type you obtained (for instance, written or verbal, and if verbal, how it was documented and witnessed). If your study included minors, state whether you obtained consent from parents or guardians. If the need for consent was waived by the ethics committee, please include this information.  If you are reporting a retrospective study of medical records or archived samples, please ensure that you have discussed whether all data were fully anonymized before you accessed them and/or whether the IRB or ethics committee waived the requirement for informed consent. If patients provided informed written consent to have data from their medical records used in research, please include this information  Once you have amended this/these statement(s) in the Methods section of the manuscript, please add the same text to the “Ethics Statement” field of the submission form (via “Edit Submission”). | 2. This study used secondary data (January 2006 – December 2020) from the Department of Agricultural Marketing (DAM), Ministry of Agriculture, Government of Peoples Republic of Bangladesh. So, the study didn't require ethical approval or respondent’s agreement. However, the data were obtained in accordance with the authority's terms and conditions and with the necessary authorizations. |
| 3. Thank you for stating the following financial disclosure:   "The authors would like to thank the Research, support and publication division of the University Grants Commission, Bangladesh (S.R. No: 6(79)/B.Mo.ko/B.O.Pro/Crop-16/2018/7032) for arranging fund to carry out this research work." Please state what role the funders took in the study. If the funders had no role, please state: ""The funders had no role in study design, data collection and analysis, decision to publish, or preparation of the manuscript.""  If this statement is not correct you must amend it as needed. Please include this amended Role of Funder statement in your cover letter; we will change the online submission form on your behalf. | 3. We have addressed the comments according to the instruction (Please see the highlighted part of the cover letter) |
| 4. Thank you for stating the following in your Competing Interests section:  "No potential conﬂict of interest was reported by the author(s)". Please complete your Competing Interests on the online submission form to state any Competing Interests. If you have no competing interests, please state "The authors have declared that no competing interests exist.", as detailed online in our guide for authors at <http://journals.plos.org/plosone/s/submit-now>   This information should be included in your cover letter; we will change the online submission form on your behalf. | 4. Thanks for your suggestions. We have replaced it. (Please see the highlighted section of the cover letter) |
| 5. In your Data Availability statement, you have not specified where the minimal data set underlying the results described in your manuscript can be found. PLOS defines a study's minimal data set as the underlying data used to reach the conclusions drawn in the manuscript and any additional data required to replicate the reported study findings in their entirety. All PLOS journals require that the minimal data set be made fully available. For more information about our data policy, please see <http://journals.plos.org/plosone/s/data-availability>. Upon re-submitting your revised manuscript, please upload your study’s minimal underlying data set as either Supporting Information files or to a stable, public repository and include the relevant URLs, DOIs, or accession numbers within your revised cover letter. For a list of acceptable repositories, please see http://journals.plos.org/plosone/s/data-availability#loc-recommended-repositories. Any potentially identifying patient information must be fully anonymized. | 5. The authors have provided the minimal data set in the supporting information file Named as Raw data (S1_Data File). |
| 6. Your ethics statement should only appear in the Methods section of your manuscript. If your ethics statement is written in any section besides the Methods, please move it to the Methods section and delete it from any other section. Please ensure that your ethics statement is included in your manuscript, as the ethics statement entered into the online submission form will not be published alongside your manuscript | 6. Thanks. We have already removed that from the other part of the manuscript. |
| **Additional Editor Comments:** | **Author’s Response** |
| 1. The gap discussed in the study is weak. author need to provide statistics about the proportion of imported onions in the total demand for onions so that this study can be deemed feasible. Further if the resources used to produce local onions are same as imported onions they definitely their market prices will move together. | 1. Thanks to the reviewers for their valuable instructions. We have provided the statistics of the demand of onion as well as the quantity of imported onion. However, the resources that are used for the production of onion may not be the same, as the weather, availability of resources are varying from country to country. So, the market prices of local and imported onion are not the same. We have improved the introduction section and research gap of the study. (Please see the Paragraph 1 and Paragraph 4 on Page 2 and 4, respectively in the Introduction section) |
| 2. What about general cost of production in the country and the exchange rate effect, which will create the difference between domestic onions and imported onions. | 2. Thank you very much for your suggestions. As the main aim of the research is to identify the asymmetric effects of price whether it is transmitted in the different level of supply chain, we did not include other variables in the model. This is the limitation of our study and we have mentioned the limitations of our study in the manuscript (Conclusion section, page 13; highlighted in yellow colour). To fulfill the research objective, we have focused only the price transmission analysis thereby we only include the price variables in the model. |
| 3. Model may have shown high coefficients because of missing variable bias. | 3. Many thanks for your comments. We have already responded this comment in previous response. Moreover, we believe that our empirical model was not suffered from the omitted variable bias because the models passed all post diagnostic test. Besides, the Ramsey RESET test revealed that the models are correctly specified. (Please see the S7_Table and Page 11 in the results and discussion section of the manuscript). |
| 4. CUSUM of squares show that model is not stable it is touching the boundaries. | 4. Thanks for the comment. As the graph of the CUSUM and CUSUM Squares are within the boundary (i.e. at 5% level), so, the models are stable in all aspect. Yes, it’s almost touching the boundaries but still within the critical line, not beyond the boundary line. (Please see the S4_Fig and S5_Fig Page 12 in the results and discussion section of the manuscript). |
| 5. Authors have not ensured that the data is normally distributed so how come the inference can be applied let alone the asymmetric effects. | 5. Thanks for your comment. From the findings of the descriptive statistics, it is clear that the data is not normally distributed (Please see the S1_Table in the supporting information file and Page 7-8 in the results and discussion part). That’s why, we can easily identify the asymmetric effects of price. Also, from the Wald test we can see that there is an asymmetry in the relationship effect so we employed the asymmetric econometric model. (Please see page 9 and also S5_Table) |
| 6. Since the model is not stable, authors cannot use the outcomes for policy implications. | 6. Many thanks for your feedback. From the above discussion, it can be said that the model is stable, So, the study provided policy guidance are valid and can be considered for the development of onion sector of Bangladesh. |
