# Supplementary material for: Spatial price transmission in the onion markets of Bangladesh: An application of NARDL approach
Source: PLoS One. 2023 Apr 19;18(4):e0284555. doi: 10.1371/journal.pone.0284555 (PMC10115283; doi:10.1371/journal.pone.0284555)
Supplement: Attachment — Submitted filename: Response to Reviewers.docx [file pone.0284555.s003.docx]

# Title: Spatial Price Transmission in the Onion Markets of Bangladesh: An Application of NARDL Approach

# Our heartfelt gratitude goes to the reviewers for their valuable feedback, which greatly aided in bolstering the quality of our paper. We have meticulously revised each segment of the work based on their constructive critique. We are optimistic that the alterations incorporated in the revised edition, as indicated below, will be well-received. All modifications have been highlighted in yellow with track changes in the manuscript.

# COMMENTS TO AUTHORS’ SPECIFIC RESPONSES

| **Reviewer 1 Comments** | **Author’s Response** |
| --- | --- |
| The paper is well written. Based on the previous comments, it seems to me that the authors have addressed them squarely. However, I have the following comments to strengthen the work:  1. The motivation for the study needs to be further strengthened. What is the reason for selection January 2006 to December 2020 for this study? | Thanks to the reviewer for his appreciation and insightful comment. Firstly, some monthly value of wholesale and retail prices are missing before 2006. Hence, we have used 15 years’ monthly data for empirical analysis because of unavailability. Another reason is that the price of onion is a serious concern in recent years. The wholesale and retail price of onion in Bangladesh is fluctuating over the ten years. Therefore, we used 15 years monthly data for our study (Kindly refer to the track changes version of the revised manuscript and locate line numbers 132-137 under the headings of data descroption). |
| 2. There is no literature review section for this study. Authors are advised to briefly review the related literature concerning the topic so that the gap the study will fill could be clear to the reader. | Thanks for your suggestion. We reviewed extensively on the prior studies of market transmission, especially agricultural product markets for determining the research gap. To keep the write up simple and easy understanding of general readers, the literature review section was incorporated in between the introduction section rather than separating it. This integration enabled easier highlight of the research gap in this aspect and addressed the importance of assessing price transmission system of imported onion and local onion in wholesale and retail markets of Bangladesh (Kindly refer to the track changes version of the revised manuscript and locate line numbers 77-100 from page 3-4 in the introduction part). |
| 3. Since the emphasis is place on NARDL (see the topic) rather than causality, I suggest that the equations concerning NARDL should come first before causality. Moreover, causal relationship equations are not numbered. The equation should be numbered so that it will be easier to refer to it. | Thanks for your suggestions. We have rearranged it. (Please see the line numbers 445-454 of the result and discussion section in the revised manuscript with track changes version) |
| 4. Why did the authors use bivariate model? There are other vital factors that can affect the interested variables. For robustness purposes, include other control variables. | Thank you very much for your valuable feedback. As the main aim of the research is to identify the asymmetric effects of price whether it is transmitted in the two stage (wholesale and retail) of onion supply chain, we did not include other variables in the model. This is the limitation of our study and we have mentioned the limitations of our study in the manuscript (Please see the line numbers 459-469 of the limitations section in the revised manuscript with track changes version). To fulfill the research objective, we have focused only the price transmission analysis thereby we only include the price variables in the model. |
| 5. Discuss clearly your major findings and compare the results with previously existing studies. | Thank you once again for your assistance. We have made every effort to ensure that the findings are presented in a clear and concise manner. We have added and modified the whole results and discussion section of the manuscript (Kindly refer to the revised manuscript with track changes version and locate line numbers 297-307 and 323-369 of the results and discussion section). However, comparing our results with previously existing studies proved challenging for the authors due to the limited literature available that only addressed domestic onion price transmission. Results of this study is annotated with available domestic vertical market studies.  We can say that the study seems to be novel as no research examined the price transmission between local and imported onion. However, we also compared with the other countries literature where available. (Please see Page 11, Line 291-292; Page 12, Line 305-307; Page 13, Line 340-342 in the revised manuscript with track changes version ). |
| **Reviewer 2 Comments:** | **Author’s Response** |
| 1. The paper needs to clarify what is the meaning of investigating the asymmetric price transmission between the wholesale and retail markets for local and imported onion. Is the objective to identify price leaderships? | The objective of investigating asymmetric price transmission is not necessarily to identify price leaderships, but rather to understand the dynamics of price transmission. By identifying the extent of asymmetric price transmission, policymakers and market players can better understand how changes in prices at one market level may impact prices at other levels and the overall functioning of the onion market. This information can help to inform pricing policies, supply chain management, and other strategies to improve market efficiency and reduce market volatility. We have clearly mentioned the aim and objectives of this study in the Introduction section (Kindly refer to the revised manuscript with track changes version and locate lines 63-66, 67-76, 106-110 and 119-123 in the introduction section). |
| 2. The authors should explain the definitions for short-run and long-run in the study. | Thanks for your feedback. We have now explained the definitions of long run and short-run price transmission. Short run is the period up to certain lag length, suppose lag 1, 2 up to 5 or 6 while the long run is the more extended period to observe the effect (Please see page 7, Line 184-186 in the revised manuscript with track changes version). |
| 3. Some discussions should be done on why the results might be difference between positive and negative shocks. | Thank you very much for your valuable instruction. We have modified the whole result and discussion sections as per the suggestions of both reviewers. Hence, we have discussed it clearly (Kindly refer to the revised manuscript with track changes version and locate lines 325-372). |
| 4. ln265 "due to the market power structure of the onion supply chain..." This part should be explained more in detail so that people not familiar with the Bangladesh onion market can understand. | Thank you once more for your kind attention. We have thoroughly revised and rephrased the results and discussion section, which has resulted in a much clearer explanation (Please refer to the line numbers 351-358 of the revised manuscript with track changes version). |
| 4. ln341 "stabilize the domestic market price of onion..." Please explain why finding asymmetric adjustment is related to stabilizing the onion market. I am not sure if asymmetric pricing always means instability of markets. I also feel most agricultural price are not stable so the policy implication of the study is a little naive. Implications should be more directly connected to the finding of the study. | I somewhat concur with your assertion that not all instances of asymmetric pricing result in market instability. However, in agricultural markets, such as the onion market, asymmetric price transmission can lead to market volatility and instability. Weather, disease, and worldwide demand and supply can cause price volatility in agricultural markets. Asymmetric price transmission between wholesale and retail marketplaces may increase volatility. When wholesale prices rise or fall, the delay or incomplete transmission of those changes to retail prices may produce market imbalances, resulting in shortages or surpluses. If imbalances persist, prices may fluctuate, making it hard for farmers, dealers, and consumers to plan and make decisions. Hence, detecting onion market asymmetry changes can reduce price volatility and improve market efficiency. In addition, we have also modified the policy implications according to the findings of our study (Please see the whole concluding remarks section i.e. line numbers 475-499 of the revised manuscript with track changes version). |
